# Perfluoroalkyl Substance Serum Concentrations and Cholesterol Absorption-Inhibiting Medication Ezetimibe

**DOI:** 10.3390/toxics10120799

**Published:** 2022-12-19

**Authors:** Ge Ma, Alan Ducatman

**Affiliations:** 1 RWJ Barnabas Health Newark Beth Israel Medical Center, Newark, NJ 07112, USA; 2 School of Public Health, West Virginia University, Morgantown, WV 26506, USA

**Keywords:** perfluoroalkyl substance, cholesterol lowering medications, ezetimibe, lipids

## Abstract

Background: Per- and polyfluoroalkyl substances (PFAS) are human-made compounds with a widespread presence in human blood and other organs. PFAS have been associated with multiple health effects, including higher serum cholesterol and LDL cholesterol. Objective: Potential population differences in serum PFAS attributable to ezetimibe, a medication that inhibits cholesterol absorption, are of interest for several reasons. The “C8” Health Project survey data from six contaminated water districts in the mid-Ohio Valley of the United States provide a wide enough range of serum PFAS and a sufficient number of ezetimibe takers to explore this topic. Methods: A total of 44,126 adult participants of the C8 Health Survey were included in the community-based study. The status of taking (1075) or non-taking of ezetimibe, alone or in combination with another lipid-lowering agent, was acquired. The geometric mean serum concentrations of the four most commonly detected serum PFAS were compared based on the status of ezetimibe use. Results: There is no significant difference in serum concentrations of perfluorohexanesulfonic acid (PFHxS), perfluorooctanoic acid (PFOA), perfluorooctanesulfonic acid (PFOS), and perfluorononanoic acid (PFNA) between ezetimibe users and non-users after adjustment for age, sex, body mass index, estimated glomerular filtration rate (eGFR), cigarette smoking, education, and average household income. Conclusion: The sterol absorption-inhibiting medication ezetimibe does not appear to affect serum PFAS concentrations. We sought but did not find direct evidence that ezetimibe could inhibit PFAS uptake nor inferential evidence that inter-individual differences in sterol absorption could provide a confounding factor explanation for the association of serum total- and LDL-cholesterol with serum PFAS.

## 1. Introduction

The purpose of this work is to investigate whether a lipid-lowering medication that interferes with the gastrointestinal absorption of cholesterol and other sterol compounds may also alter the human absorption of per-and polyfluoroalkyl substances (PFAS). PFAS are exclusively manmade compounds with numerous industrial and consumer uses, providing nonstick and grease- or water-resistant properties to many products such as cookware, fabrics and upholstery, food packaging, and friction-reducing surface coatings [1]. Environmentally persistent and important PFAS pollutants include perfluoroalkyl acids such as perfluorooctanoic acid (PFOA), perfluorooctane sulfonate (PFOS), perfluorohexane sulfonic acid (PFHxS), and perfluorononanoic acid (PFNA). These have long serum half-lives in humans and have been detected consistently in human serum [2,3,4,5]. Studies across different populations and with both cross-sectional and longitudinal study designs have found that PFAS serum concentrations or modeled total exposure levels are deleteriously associated with several lipid outcomes, including increased total- and low-density lipoprotein (LDL) cholesterol in adults, pregnant women, children, and adolescents [6,7,8,9,10,11,12,13,14,15]. These and other studies have indicated that the adverse association of serum PFAS to lipid profiles has been robust to adjustment for factors that are known to partially predict PFAS serum concentrations, such as age, sex, socioeconomic status, smoking, and body habitus. 

There is public interest in measures, including pharmaceutical agent interventions, which could decrease internal exposure to PFAS or enhance PFAS excretion, as well as recognition that the activity of organic anion transporters related to PFAS excretion in experimental studies [16]. A 52-week randomized clinical trial among 285 Australian firefighters showed that 6-week serial plasma donations led to small but significantly reduced serum PFOS and PFHxS compared to controls, while 12-week serial blood donations decreased PFOS only [17,18]. In a large community study, the 23 participants who reported taking the organic anion transporter (OAT) inhibiting agent probenecid had nonsignificant increases in serum PFAS, whereas 36 who reported taking bile acid sequestrant cholestyramine experienced markedly and significantly lower serum PFHxS, PFOA, PFNA, and especially PFOS, compared to the control population [19]. The cholestyramine results were consistent with the results of a previous, informal clinical trial in a heavily exposed family [20], showing that interruption of bile acid recirculation can reduce PFAS absorption and enhance excretion. However, the authors of the cross-sectional community study cautioned that the strong results did not imply knowledge about the comparative risks and benefits of cholestyramine use for reducing internal PFAS in individual patients [19]. 

Noncausal explanations for the association of PFAS exposure to disrupted human cholesterol metabolism have been studied and are relevant to discussions of PFAS absorption, distribution, exposure, or excretion. Butenhoff and colleagues investigated but did not find evidence for the plausible noncausal hypothesis of serum distribution of PFOA and PFOS into lipoprotein fractions as an explanation for the association [21]. Similarly, higher serum lipid concentrations did not reduce excretion or enhance serum retention of PFOA [22]. Based on the effects of fiber on enhancing fecal excretion and prior PFAS research [23,24], Dzierlenga and colleagues investigated the specific role of dietary fiber in reducing serum PFAS. Their investigation found the expected inverse relationship between fiber intake and serum PFAS in NHANES data and plausibly attributed the association to enhanced PFAS excretion [25]. In addition, they and others have questioned if dietary differences could be important to understand whether the association of serum PFAS to cholesterol levels is causal [25,26]. However, this source of confounding is not very plausible for the association of serum PFAS to cholesterol, as fiber reliably lowers serum cholesterol as well as serum PFAS [27], and similar dose-response curves for unfavorable lipid outcomes of PFAS exposure are also seen in large-scale studies of PFAS water-exposed communities on two continents [2,14,28]. There is no reason to think that fiber exposure differs in communities with drinking water exposure to PFAS. Recently, authors who raised the question concerning confounding by fiber intake performed a Bayesian analysis in NHANES data showing that fiber intake is not an important confounder of the association between PFAS exposure and cholesterol [29]. 

There have also been questions concerning whether the increase in serum lipids seen in PFAS exposure could be related to upregulated fatty acid transport genes or downregulated reverse transport following PFAS exposure [30,31]. If shared pathways of uptake for both PFAS and cholesterol or cholesterol precursors are important, it is possible that the relationship of PFAS to clinical biomarkers such as total and LDL cholesterol is mediated by precursor uptake and potentially noncausal. Inter-individual differences in lipid and PFAS absorption and excretion remain of interest, and could be related to a combination of causal and noncausal explanations for the associations of serum PFAS to deleterious lipid profiles, and so far, convincing noncausal explanations have not been found. 

We hypothesized that a medication that specifically decreases gut intake of cholesterol and sterol precursors might also inhibit PFAS absorption. This outcome, if supported by data, would be of interest because it could provide another useful candidate for enhanced PFAS excretion and could also provide an indirect way to evaluate whether inter-individual differences in sterol uptake could explain the association of PFAS exposure to lipid outcomes. Ezetimibe is a cholesterol-lowering agent that blocks the absorption of sterols, including cholesterol in the small intestine, primarily by inhibiting Niemann-Pick C1-like 1 (NPC1L1) protein and also potentially reducing flux in the bile [32,33,34,35]. It prevents cholesterol uptake into enterocytes of the small intestine. Gene polymorphisms that affect nuclear receptors may influence many pathways, including NPC pathways, in the associations of PFAS and serum lipid levels [36]. A US National Library of Medicine dataset addresses polymorphic variations in NPC1L1 and effects on lipid levels (https://www.ncbi.nlm.nih.gov/gene/29881 accessed on 2 December 2022), with different polymorphisms having different effects on serum cholesterol, but there are no data about PFAS effects on NPC1L1 or NPCLI effects on serum PFAS. Nevertheless, inferential yet strong human evidence supports a role for agents that may inhibit enterohepatic recirculation and reuptake of PFAS as lowering serum PFAS levels [19,37], but it is unclear if an agent such as ezetimibe that reduces sterol uptake in the gut and in the biliary tree would also reduce serum PFAS. We investigated whether an agent that selectively blocks sterol absorption would also interfere with PFAS absorption by comparing serum PFAS in ezetimibe users and non-users in a large population with a wide range of serum PFAS exposure.

## 2. Methods

### 2.1. Study Participants

Data on the population measures were obtained from the C8 Health Project. The purpose of the project related to serum levels of PFOA in a community with PFOA water contamination and health outcomes; the methods are described in detail in [9,38]. Briefly, funding for eligibility for participation was provided under the settlement of a class action lawsuit in a region where 6 water districts in two US Appalachian states were variably contaminated with PFOA (≥0.05 ng/mL), resulting in human internal contamination levels ranging from contemporary US population background levels of serum PFOA contamination in some participants to very high levels for PFOA contamination in others [38]. Other serum PFAS such as PFOS and PFHxS distributed at roughly NHANES population levels for the 2005–2006 contemporary data (NHANES reports serum PFAS in a representative sample of the US population over time [5], facilitating such comparisons). Subjects were eligible if they could document that they had lived, worked, or gone to school in a contaminated water district for at least 1 year. An estimated 81% of the 2005–2006 residents participated in the C8 Health Project [14]. The survey collected demographic, anthropogenic, and health data from 69,030 residents of all ages, including 44,126 adults with completed pharmaceutical status data and the four most commonly detected serum PFAS (PFOA, PFOS, PFHxS, PNFA). Other PFAS analytes were measured in the survey, but excessive numbers of undetected values diminish the evaluative potential of all but the four analytes for the purposes of this paper concerning PFAS serum levels and medication effects on PFAS serum concentrations. 

### 2.2. Outcomes of Interest and Laboratory Methods

The main outcome reported in this paper was the usage of the cholesterol-lowering medication ezetimibe and serum PFAS. Medication use for ezetimibe and other pharmaceutical agents was acquired from the survey questionnaire and based upon self-report. The analytical method of PFAS used in this study is based upon methods that have been described previously [39] and also described for this population specifically [38,40]. Briefly, the method utilized solid-phase protein precipitation extraction combined with reverse-phase high-performance liquid chromatography/tandem mass spectrometry. PFAS analytes were detected using a triple quadrupole mass spectrometer in selected reaction monitoring mode for the m/z transition of each PFAS and the specific ^13^C surrogate. The detection limit was 0.5 ng/mL. Clinical laboratory and PFAS biomarker exposure results were automatically uploaded into a secure server.

### 2.3. Statistical Analysis

The outcomes were serum concentrations of PFHxS, PFOA, PFOS, and PFNA. These four analytes were above the detection limit in >97% of the population. When a PFAS was not detected, we assigned a value of 0.25 ng/mL, half the value of the detection limit, consistent with the practice in previous reports for this population [40]. Covariates used for adjustment were chosen because of their established relationship to PFAS serum concentrations, consistent with many other publications. Covariates included age, sex, body mass index (BMI), education (less than high school, high school, some college, bachelor’s degree or higher), income (gradations provided in Table 1), and smoking (current, former, never). In addition, we adjusted for the estimated glomerular filtration rate using the Modification of Diet in Renal Disease (MDRD) equation [41]. Although participant lipid levels are not an outcome variable for this effort, ezetimibe use is predicated upon abnormal lipid profiles, which can entail more than one drug treatment. Therefore, we created a sensitivity test examining effects limited to takers of ezetimibe only in order to evaluate if the results were influenced by other lipid-lowering medications. Because of the consistent relationship of serum PFAS to serum total and LDL cholesterol [42], including in this population specifically [14], we also compared ezetimibe users only to those also taking other lipid-lowering medications in order to avoid confounding by indication. The small number of cholestyramine takers were excluded because of the known serum PFAS-lowering effect of this agent [19].

## 3. Results

Table 1 summarizes the descriptive statistics. In a population of 44,126 adults eligible for inclusion in this study, a total of 1075 reported taking ezetimibe. This medication use rate is consistent with national pharmacy benefit surveys from approximately the same period [43]. Ezetimibe users were older and less likely to smoke compared to the full population, but otherwise similar to non-users. Baseline PFAS characteristics are presented, showing that serum PFAS was consistently higher in ezetimibe users. 

Outcome variables for the fully adjusted model, including geometric mean serum concentrations of PFHxS, PFOA, PFOS, and PFNA are provided in Table 2, which also provides the serum comparisons between ezetimibe users and non-users in both unadjusted and fully adjusted models with 1 set as the referent value. Significant positive differences were seen for PFOA (1.08, 95% C.I. 1.04, 1.15, *p* = 0.05) and PFHxS (1.09, 95% C.I. 1.04, 1.15, *p* < 0.01) between ezetimibe users compared to non-users before adjustment. These differences were no longer statistically significant after adjustment (all confidence intervals spanned 1.00, Table 2), and the directions of differences appeared random and not related to whether the compound was a carboxylate or a sulfonate. 

### Sensitivity Testing

Table 3 shows the comparison between ezetimibe takers who were and were not taking statins, as well as statin users who were not taking ezetimibe, compared to the use of other lipid-lowering agents. We introduced these comparisons in order to address confounding, including confounding by indication, to the degree possible. Ezetimibe alone is associated with a higher serum PFAS (except for PFNA), and after adjustment, the differences between ezetimibe users and statin users and other lipid-lowering agent users are not significant, except that statin users who are not using ezetimibe have significantly higher serum PFOS (only) than the referent group taking other lipid-lowering agents. 

## 4. Discussion

Based on our experience with strongly lower serum PFAS associated with the use of cholestyramine [19], as well as possible genetic evidence suggesting ezetimibe-relevant pathway effects [36], our study sought and did not find evidence that ezetimibe might affect serum PFAS levels. Ezetimibe takers had serum PFAS no different than other participants who took and who did not take lipid-lowering agents. By inference, ezetimibe does not affect gut PFAS absorption. Ezetimibe is known to lower serum cholesterol [44], and our data shows that it is not associated with lower serum PFAS levels.

The drug activity of ezetimibe, which lowers serum cholesterol but not serum PFAS, and cholestyramine, which lowers both serum cholesterol and serum PFAS (and especially PFOS), are similar but not the same. Cholestyramine sequesters bile acids and limits their reabsorption. Ezetimibe inhibits the absorption of neutral sterol compounds such as cholesterol and decreases the amount of cholesterol normally available to liver cells. Ezetimibe blocks the critical mediator of cholesterol absorption, the NPC1L1 protein in the gastrointestinal tract epithelial cells, as well as in hepatocytes, and interrupts a Caveolin 1-Annexin A2 complex involved in trafficking cholesterol [35]. By inference, the ezetimibe data in this paper do not provide support that the repeatedly demonstrated relationship between PFAS and serum lipids in humans might be due primarily to inter-individual differences in the efficiency of human sterol uptake in the gut and liver, where ezetimibe operates, such as genetic or other differences that might affect serum uptake of PFAS and cholesterol building blocks simultaneously. However, the generalizability of our findings may or may not be limited to ezetimibe’s primary mode of action, the inhibition of NPC1L1. 

Strengths of this study include its large size and that it stems from a population in which the association with total- and LDL-cholesterol is already characterized. Our study also has important limitations, notably the use of prevalent data. We are reporting associations with biomarkers of exposure at a single point in time, which is a strength for evaluating the association but a weakness for asserting causation. This weakness is mitigated but not fully vanquished by the long half-lives of the four compounds studied, and of course, the data do not support a causal link of ezetimibe use with serum PFAS. Another weakness is the necessary use in this and other surveys of questionnaire data for medication use. Our results depend on report reliability and interpretation. The multiple names and combinations of medications are a real challenge, as are spellings by participants, which we considered, but there is room for error in recording and interpreting. The initiation dates of medications are not in the dataset. These factors could be a source of bias if accurate medication recording and designations by survey participants are somehow linked to serum PFAS concentrations. A study-specific concern we have attempted to address is “confounding by indication”. Ezetimibe is prescribed for hyperlipidemia, and elevated serum cholesterol is already associated with PFAS exposure in multiple populations from around the world [8,9,10,11,12,13,42,45,46], including the adult C8 population [14,28], which is the subject of this study. We, therefore, chose a sensitivity test comparing ezetimibe users alone to those using ezetimibe and other lipid-lowering agents in combination, as well as to those using other agents without the use of ezetimibe. The finding that ezetimibe is not an effective mediator that lowers serum PFAS is clear from the data. An important limitation is the scope of our investigation. The ezetimibe data in this paper argue against a correlated uptake explanation for the association of serum PFAS with serum total and LDL cholesterol, but the data cannot rule out correlated PFAS and lipid-precursor uptake that may proceed by mechanisms that are different from the specific mechanisms affected by ezetimibe. This finding is consistent with a previous evaluation of ezetimibe in a smaller population with a narrower range of exposure [26].

The incidental finding in the fully adjusted data is that serum PFOS (only) is slightly higher in statin users than in users of other lipid-lowering agents. This deserves some discussion and has important limitations in our analysis. It arises from multiple comparisons of a non-hypothesis-based analysis, albeit the finding also appears to have support from a smaller study in previous literature [26]. It also warrants consideration for confounding by indication, which is one of the reasons we compared it to other lipid-lowering agents. PFAS are associated with higher serum cholesterol across populations, and statin adapters may have had higher cholesterol than users of other lipid-lowering medications, especially in 2005–2006 when our survey data were collected. Importantly, the finding may or may not provide clues concerning PFAS but should not be confused with the possibility that PFAS associations with serum cholesterol are confounded by the effects of lipid-lowering agents. The association of PFAS exposure to higher cholesterol is present in multiple populations when lipid medication users are excluded, such as in this same population [14] and in the large Veneto study [47]. Nevertheless, we hope other investigators will address statins and other medication associations in longitudinal, hypothesis-based studies. They may provide additional clues to help us understand the activity of PFAS. 

Parallel data show that the hepatoxicity of PFAS is not limited to higher cholesterol; reviews show that higher serum transaminases, and especially higher alanine aminotransferase (ALT), are seen in multiple populations based on PFAS exposure. Furthermore, there is ample experimental evidence that PFAS are hepatotoxic and cause lipid accumulation in liver cells [42,48]. This study provides some additional inferential data about the role of absorption concerning these outcomes and does not suggest a reason for thinking that inter-individual differences explain the PFAS–lipid or PFAS–transaminase associations. Based on the inability to find other causes for the association in two decades of research, we suspect the association between PFAS and cholesterol and LDL cholesterol is causal and commend the continued efforts to better understand its origins. 

## 5. Conclusions

Our study demonstrated no association between cholesterol-lowering medication ezetimibe usage and serum PFAS levels. Ezetimibe usage is not a confounding factor to serum PFAS. 

## Figures and Tables

**Table 1:** Baseline characteristics of the study population *.

Characteristics	Whole Cohort	Ezetimibe Users	*p*-Value
	(*n* = 44,126)	(*n* = 1075)	
Age (years)	51.15 ± 13.25	59.67 ± 10.76	<0.001
Sex (Female, %)	23,198 (52.27)	529 (49.21)	0.015
Body mass index (kg/m^2^)	29.20 ± 7.84	30.53 ± 5.90	<0.001
Estimated glomerular filtration rate ^a^	75.83 ± 16.93	70.45 ± 22.33	<0.001
Current Cigarette Smoking (Yes, %)	10,718 (24.18)	182 (16.96)	<0.001
Education (%)			
<12 years	5252 (11.90)	141 (13.21)	0.113
HS Diploma or GED	19,095 (43.25)	499 (46.77)	0.02
Some College	13,861 (31.39)	306 (28.68)	0.02
Bachelor Degree or Higher	5944 (13.46)	121 (11.34)	0.018
Average Household Income (%)			
<$10,000	3191 (7.89)	64 (6.70)	0.055
$10,001–$20,000	5730 (14.16)	134 (14.03)	0.386
$20,001–$30,000	6475 (16.00)	158 (16.54)	0.492
$30,001–$40,000	5895 (14.57)	150 (15.71)	0.284
$40,001–$50,000	4917 (12.15)	132 (13.82)	0.121
$50,001–$60,000	4242 (10.48)	95 (9.95)	0.198
$60,001–$70,000	3368 (8.32)	73 (7.64)	0.151
>$70,000	6641 (16.41)	149 (15.60)	0.14
PFHxS ng/mL) †	2.93 ± 2.23	3.19 ± 2.21	<0.001
PFOA (ng/mL) †	35.83 ± 3.62	38.67 ± 3.69	<0.001
PFOS (ng/mL) †	19.75 ± 2.04	20.70 ± 2.19	<0.001
PFNA (ng/mL) †	1.37 ± 1.65	1.38 ± 1.64	0.843

* Data presented are number (percentages) or mean values ± standard deviation (SD), as appropriate for the variable; ^a^ mL/min/1.73 m^2^; † geometric mean values (SD).

**Table 2:** Fully adjusted association between ezetimibe use and PFAA levels.

	Sample Size	Geometric Mean (SD)	Unadjusted Geometric Mean Ratio (95% CI)	Multivariable Adjusted * Geometric Mean Ratio (95% CI)
PFHxS				
Ezetimibe non-users	43,054	2.92 (2.23)	1 (referent)	1 (referent)
Ezetimibe users ^+^	1072	3.19 (2.21)	1.09 (1.04, 1.15)	1.03 (0.98, 1.08)
*p*-value			0.0004	0.24
PFOA				
Ezetimibe non-users	43,054	35.76 (3.62)	1 (referent)	1 (referent)
Ezetimibe users ^+^	1072	38.67 (3.69)	1.08 (1.00, 1.17)	0.95 (0.87, 1.03)
*p*-value			0.05	0.18
PFOS				
Ezetimibe non-users	43,054	19.73 (2.03)	1 (referent)	1 (referent)
Ezetimibe users ^+^	1072	20.70 (2.19)	1.05 (1.01, 1.10)	0.96 (0.92, 1.00)
*p*-value			0.03	0.06
PFNA				
Ezetimibe non-users	43,054	1.36 (1.65)	1 (referent)	1 (referent)
Ezetimibe users ^+^	1072	1.38 (1.64)	1.01 (0.98, 1.05)	1.02 (0.98, 1.05)
*p*-value			0.36	0.34

* Adjusted for age, sex, body mass index, estimated glomerular filtration rate, cigarette smoking, education, and average house income; + include single ezetimibe user and ezetimibe combined with other classes of cholesterol medications.

**Table 3:** Fully adjusted association between different cholesterol medications and PFAAs level.

	Sample Size	Geometric Mean (SD)	Unadjusted Geometric Mean Ratio (95% CI)	*p*-Value	Multivariable Adjusted * Geometric Mean Ratio (95% CI)	*p*-Value
PFHxS						
Ezetimibe users	401	3.22 (2.17)	1.11 (1.00, 1.22)	0.0403	1.08 (0.98, 1.20)	0.1342
Statin users	6175	3.20 (2.23)	1.10 (1.04, 1.17)	0.0014	1.07 (1.00, 1.13)	0.0438
Ezetimibe and Statin users	671	3.18 (2.23)	1.09 (1.01, 1.19)	0.0353	1.08 (0.99, 1.18)	0.0839
Other cholesterol medication user	800	2.91 (2.19)	1 (referent)		1 (referent)	
PFOA						
Ezetimibe users	401	43.49 (3.53)	1.18 (1.01, 1.38)	0.0370	1.17 (0.99, 1.38)	0.0652
Statin users	6175	42.06 (3.67)	1.14 (1.04, 1.26)	0.0068	1.11 (1.00, 1.22)	0.0517
Ezetimibe and Statin users	671	36.05 (3.77)	0.98 (0.86, 1.12)	0.7471	0.96 (0.83, 1.11)	0.6104
Other cholesterol medication user	800	36.07 (3.62)	1 (referent)		1 (referent)	
PFOS						
Ezetimibe users	401	19.65 (2.31)	1.01 (0.93, 1.10)	0.8109	1.00 (0.91, 1.10)	0.9562
Statin users	6175	22.91 (2.04)	1.18 (1.12, 1.24)	<0.0001	1.14 (1.08, 1.21)	<0.0001
Ezetimibe and Statin users	671	21.36 (2.11)	1.10 (0.02, 1.18)	0.0144	1.08 (1.00, 1.17)	0.0645
Other cholesterol medication user	800	19.44 (2.36)	1 (referent)		1 (referent)	
PFNA						
Ezetimibe users	401	1.36 (1.70)	0.99 (0.93, 1.05)	0.6866	0.98 (0.92, 1.04)	0.4468
Statin users	6175	1.40 (1.64)	1.02 (0.98, 1.06)	0.3131	1.02 (0.99,1.06)	0.2214
Ezetimibe and Statin users	671	1.40 (1.60)	1.02 (0.97, 1.07)	0.4664	1.02 (0.97,1.08)	0.4495
Other cholesterol medication user	800	1.37 (1.63)	1 (referent)		1 (referent)	

* Adjusted for age, sex, body mass index, estimated glomerular filtration rate, cigarette smoking, education, and average house income.

## Data Availability

These data are hosted at West Virginia University under a settlement agreement.
